# Arctic megaslide at presumed rest

**DOI:** 10.1038/srep38529

**Published:** 2016-12-06

**Authors:** Wolfram H. Geissler, A. Catalina Gebhardt, Felix Gross, Jutta Wollenburg, Laura Jensen, Mechita C. Schmidt-Aursch, Sebastian Krastel, Judith Elger, Giacomo Osti

**Affiliations:** 1 Alfred-Wegener-Institut Helmholtz-Zentrum für Polar- und Meeresforschung, Bremerhaven, Germany; 2 Christian-Albrechts-Universität, Kiel, Germany; 3 GEOMAR Helmholtz-Zentrum für Ozeanforschung, Kiel, Germany; 4 CAGE - Centre for Arctic Gas Hydrate, Environment and Climate, Department of Geology, UiT-The Arctic University of Norway, Tromsø, Norway

## Abstract

Slope failure like in the Hinlopen/Yermak Megaslide is one of the major geohazards in a changing Arctic environment. We analysed hydroacoustic and 2D high-resolution seismic data from the apparently intact continental slope immediately north of the Hinlopen/Yermak Megaslide for signs of past and future instabilities. Our new bathymetry and seismic data show clear evidence for incipient slope instability. Minor slide deposits and an internally-deformed sedimentary layer near the base of the gas hydrate stability zone imply an incomplete failure event, most probably about 30000 years ago, contemporaneous to or shortly after the Hinlopen/Yermak Megaslide. An active gas reservoir at the base of the gas hydrate stability zone demonstrate that over-pressured fluids might have played a key role in the initiation of slope failure at the studied slope, but more importantly also for the giant HYM slope failure. To date, it is not clear, if the studied slope is fully preconditioned to fail completely in future or if it might be slowly deforming and creeping at present. We detected widespread methane seepage on the adjacent shallow shelf areas not sealed by gas hydrates.

Subaquatic mass movements such as large-scale slides are among the major geohazards since they can trigger ocean basin-wide tsunamis. Many such slides have been reported from prehistoric times^1^,^2^, and historic records^3^. Mega-scale and multi-phase slide complexes are described from many areas worldwide, often located in areas of high risk because of dense populations. The Arctic Ocean is also influenced by slope instabilities (e.g., refs 4 and 5), however, so far, just one megaslide has been described^6^,^7^,^8^. Methane ebullition and the presence of gas hydrates have been reported from the Laptev and East Siberian Seas^9^,^10^ but not yet from the shelves to the north of Svalbard. The Arctic changes rapidly in response to global warming and this change is expected to accelerate^11^. The present day warming is amplified in the Arctic Ocean by a factor of 2 to 3 for air and water temperatures^12^. This has led to a 1 K temperature rise of advected bottom waters over the last three decades^13^,^14^. In places where warmer bottom waters reach the seafloor, methane ebullition indicates dissociating gas hydrates^10^,^15^,^16^,^17^. Although the potential extent of hydrate dissociation is controversially discussed, rising bottom water temperatures and potential gas hydrate dissociation challenge the stability of future Arctic Ocean’s continental slopes^18^,^19^. Thus, facing the increasing hydrocarbon exploration of this remote area, an assessment of the potential hazard and coherent risk of slope failure in the Arctic Ocean is of high importance.

The glacier-fed, siliclastic continental margin north of Svalbard is known for the giant Hinlopen/Yermak Megaslide (HYM) that was discovered at the mouth of the Hinlopen cross-shelf trough using bathymetry, backscatter and seismic data^6^, Fig. 1. An amphitheatre-shaped slide scar area with head and sidewalls up to 1600 m high indicates that at least 1250 km^3^ of shelf sediments were excavated, and up to 2400 km^3^ of sediment were finally involved in the slide^7^,^8^. The slide affected an area of well over 10000 km^2^; the run-out distance exceeds 300 km. Large blocks with lateral dimensions of up to 4 km and taller than 300 m can be observed in the depositional area. The failure event was dated to 30 cal kyr B.P.^8^. In contrast to other megaslides, e.g., the Storegga slide (STS)^20^,^21^, the Hinlopen shelf failed coincidently with rapidly falling sea-level during the last glaciation^22^. To date, there has been no clear evidence for the presence of gas hydrates, free gas or degassing features, which led Winkelmann and Stein^22^ to argue that hydrate dissociation and gas overpressure are not among the main preconditions for slope failure initiation. Instead, they favoured tectonic control, related to the development of a forebulge as the glaciation intensified. Whereas a huge parts of the Northern Svalbard slope failed completely during the HYM event, nearby slope segments seem to be intact on a first glance. However, existing sparse hydro-acoustic, low-resolution seismic, and core data^6^,^23^ already show that the lower slope of the western Nordaustlandet shelf experiences soft sediment deformation, most probably following the HYM. These first signs of slope instability motivated the acquisition of new bathymetric, hydro-acoustic and high-resolution seismic data in 2013.

## Results

### Bathymetry

**Figure 1:**
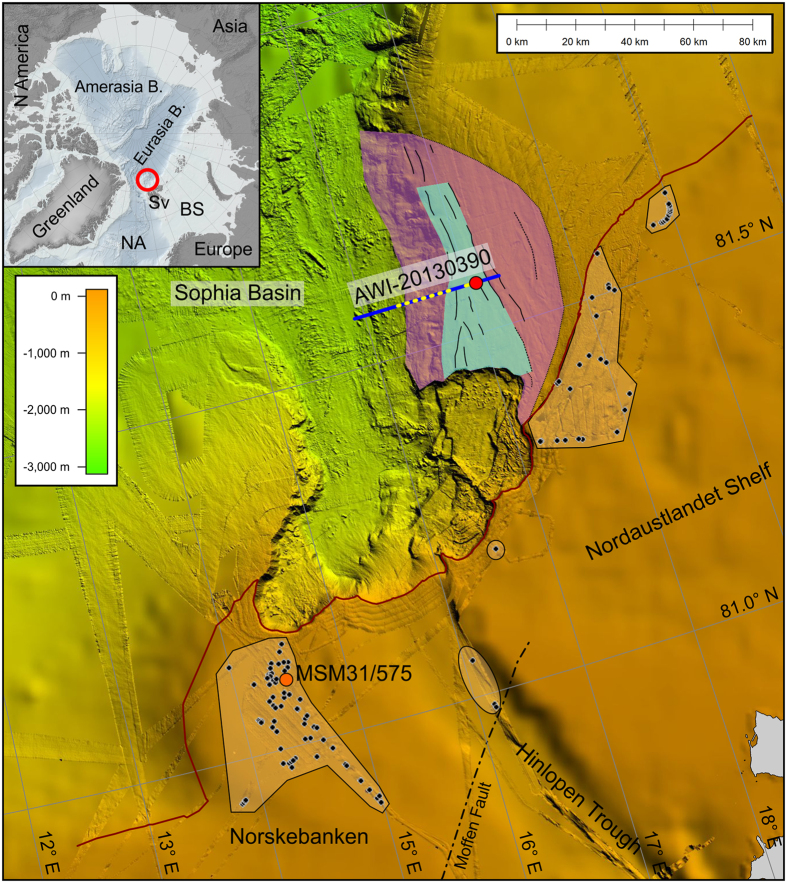
Compiled seafloor topography (sources: refs 7, 8 and 44, new data) illustrates the Hinlopen/Yermak Megaslide as well as the intact slope north of it. Inset: Study area within the Arctic Ocean. B, Basin; BS, Barents Sea; NA, North Atlantic; Sv, Svalbard. Black dots, acoustic gas flare locations; red dots, buried gas vent locations; orange dot, sample location; black lines, curvilinear features (steps) at the seafloor; blue line, high-resolution seismic profile; yellow dotted lines, surface projection of observed bottom-simulating reflections; red line, 300 m isobath; turquoise area, extrapolated surface of incipient slide block; violet area, slope affected by instability; orange areas, areas of mapped acoustic gas flares. We used Global Mapper (V16.1) to create the map. For uninterpreted seafloor topography see Fig. S1.

The main area of the HYM and the lower part of the adjacent western Nordaustlandet slope was already mapped in the past^7^,^8^,^23^. Our new bathymetric data set covers now almost the complete western Nordaustlandet slope from the shallow shelf down to the deep sea with a maximum resolution in the order of 20–50 m (Fig. 1) and significantly complements the existing bathymetric data. The new data show that the western Nordaustlandet shelf slope resembles a half-bowl. Furthermore, we observe curvilinear features at the seafloor within this half-bowl, which are actually steps with up to 40 m topography and lengths of up to >32 km.

### Seismic Reflection Data

**Figure 2:**
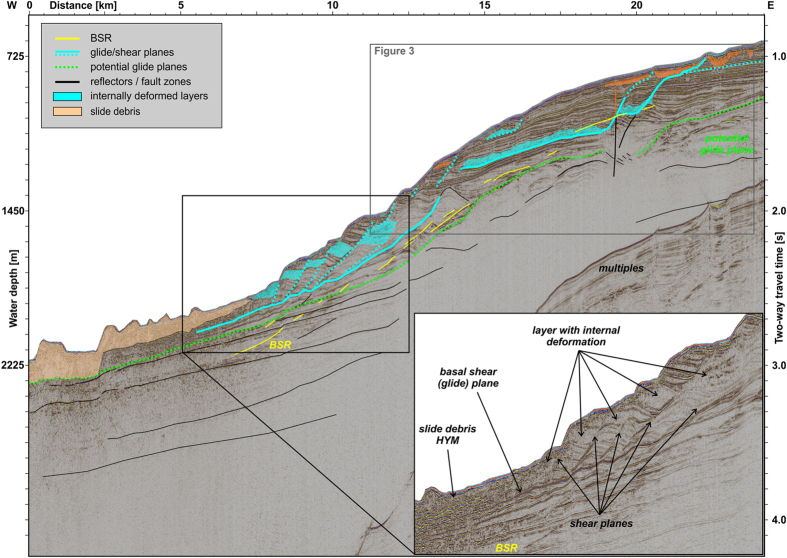
Interpreted seismic profile AWI-20130390. The slide debris at the foot of the slope stems from the Hinlopen/Yermak Megaslide. BSR, Bottom simulating reflector. For uninterpreted version see Fig. S2.

**Figure 3:**
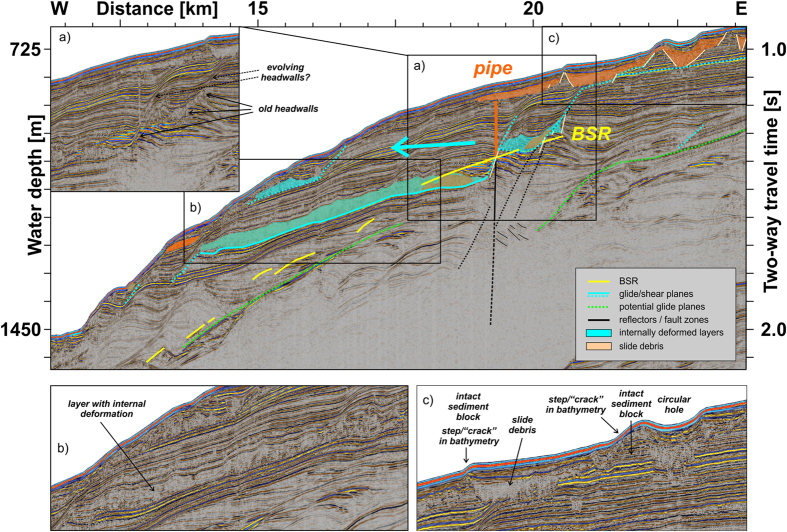
Interpretation of seismic profile AWI-20130390. The turquoise arrow should indicate that the slope started to move/slid westward. BSR, Bottom simulating reflector. For uninterpreted version see Fig. S3.

Seismic profile AWI-20130390 (Figs 2 and 3) images well-stratified contourite deposits intercalated with glacial deposits beneath the western Nordaustlandet slope, in much higher resolution than any profiles published before^6^,^7^,^23^. Reflector geometry at the lower slope (Fig. 2) can at first glance be interpreted as sediment wave deposits from contour currents. However, the seismic data also clearly show that shear planes evolved out from the pre-existing sedimentary geometry, supporting the first low-resolution observations made by Cherkis *et al*.^6^ and Winkelmann *et al*.^23^.

For the first time, the new seismic data image prominent bottom-simulating reflections (BSR) marking the base of the gas hydrate stability zone (GHSZ) at approximately 240 m (0.26 s TWT) below the seafloor at the upper slope (Fig. 2). The BSR is a clear evidence for the presence of gas hydrates just a few kilometres north of the HYM sidewalls. High amplitude reflections beneath the GHSZ proof the presence of free gas within the sediments. Weaker BSRs are imaged further downslope, at 1000 to 1900 m water depth (Fig. 2). At profile kilometre 19.3 the seismic profile reveals a prominent vertical pipe connecting the gas reservoir beneath the GHSZ with the shallow sub-seafloor (Fig. 3). This pipe, comparable to pipes near the STS^24^, is a structure formed during a sudden gas release and so documents an era of over-pressure in the gas reservoir (e.g., ref. 25). Both, the reservoir and pipe, might be located above a deeper-reaching fault zone, but the data set is not conclusive about it. The pipe terminates at the overlying bottom of an acoustically chaotic body, most probably from a minor debris flow origin that in turn is overlain by ~20 m of well-stratified hemipelagic sediments. At the top of the debris flow, a tiny amplitude anomaly indicates, however, that the gas also penetrated further upwards. Despite this, the pipe is not expressed at the seafloor, indicating that it is sealed by the overlying low-permeable (fine-grained) contourites or, more probably, has been inactive since the time of the debris flow emplacement. We do not observe any acoustic flares in the water column at this site and its vicinity, indicating a presently intact sealing.

As already mentioned above, we image acoustically chaotic bodies in the shallow sub-seafloor at profile kilometres 19 to 24 (Fig. 3), which are most probably built from slide debris of minor, secondary slope failures. But, there still exist apparently intact sediment blocks in-between the slide debris. The positions of these blocks seem to be associated with the observed curvilinear features (steps) in the bathymetry (see above), providing information on the lateral extent of the apparently intact sedimentary blocks.

In close spatial relation to the pipe and present-day BSR we observe a zone of slightly- to strongly- deformed sediments on top of a strong reflecting layer between profile kilometres 14 and 19 (turquoise area in Fig. 3). Also the layers immediately above show indications for folding. We interpret this internal deformation as an expression of the overlying sedimentary block having slid along a glide plane at the strong reflector. Discontinuities in reflector geometry between profile kilometres 19 and 21 indicate the existence of old headwalls that were later covered by contourite drift deposits. Sediments with an internal seismically chaotic facies in close vicinity to these old headwalls might represent the old slide debris. But we think that the turquoise marked zone essentially represents a younger glide zone that likely evolved at or close to an older failure surface. Sediments within the internally deformed layers might partially consist of old slide debris and interlayered contouritic/glacigenic sediments.

Out from the fault zone imaged at profile kilometre 19.3 (Fig. 3), secondary faults seem to spread eastward upwards through the mostly contouritic sedimentary cover. Deduced from the continuity of seismic reflectors, sediments did not yet loose coherence completely, but deformation and shearing already occurred along the evolving faults. These zones of deformation (evolving shear bands) are in close spatial relation to the older pre-existing headwalls. Further up-slope the zones of deformation are connected to the intact sedimentary blocks and minor slope failure events, indicating a causal relation.

### Water column Imaging

**Figure 4:**
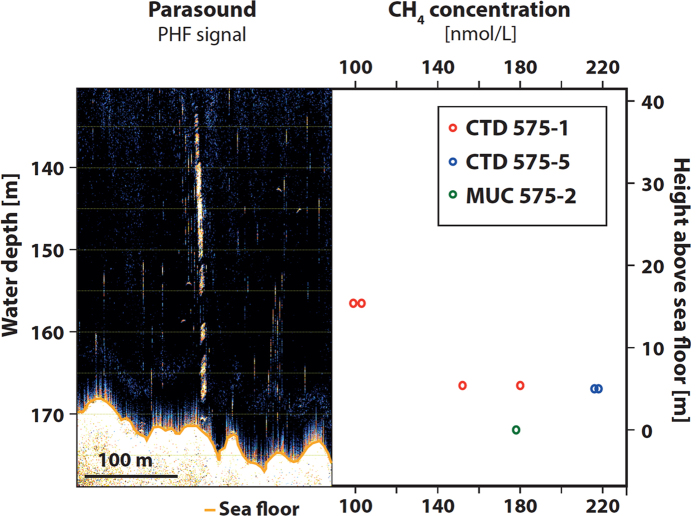
Gas flare sampling at station MSM31/575. Left: 18 KHz Parasound Primary High Frequency (PHF) image of the acoustic flare. Right: methane concentration in water samples taken close to the seafloor.

Along most of our tracks to the north of Svalbard, we observed acoustic flares at depths almost exclusively shallower than 300 m in the water column (Fig. 1, Table S4). They occur immediately to the south of the HYM main headwalls but also on the Nordaustlandet shelf. We attribute these flares to widespread methane venting and proofed this assumption by sampling bottom water at venting site MSM31/571 (Fig. 4, Table S5). Methane concentrations of up to 218 nmol/L and 178 nmol/L were measured in samples collected from Niskin bottles of two CTD casts and from water covering the sediments in tubes from a multicorer cast. These values match those from CTD casts at methane ebullition sites off West Spitsbergen, and are significantly enriched against background values of ~10–13 nmol/L^26^.

During the expedition we observed no flares in water deeper than 300 m, the approximate depth at which the base of gas hydrate stability zone (GHSZ) intersects the seafloor, assuming a mean seabed temperature of 0 °C^9^,^27^. Since no pockmarks are observable in our bathymetric data at least close to flare locations (resolution 20–50 m), we conclude that the current venting is still diffuse and methane emanates from areas without gas hydrate sealing. Whereas, in areas where we observe BSRs, the sealing by gas hydrates seems to be still intact at present.

## Discussion

Submarine slope failures and their mechanisms are controlled by contrasts in gravitational stress and sediment weakness^28^. Especially, contourite deposits as they also occur at the western Nordaustlandet slope are prone to instability^21^,^29^,^30^. Their usually fine-grain size, high sedimentation rates, high grade of sorting, low permeability und high pore water content cause a generally low shear strength and therefore favour the formation of over-pressured gliding planes (e.g., refs 31 and 32). Especially at high latitude margins, rapid accumulation of glacigenic sediments adds to the preconditioning factors of potential failure^33^, as it was for sure the case for the main HYM and might also partly be valid for the studied slope segment. There is still an ongoing discussion if gas hydrates within the slope sediments are a stabilizing factor or actually responsible for instabilities due to strain softening^34^. However, there is a general agreement that gas hydrate dissociation, either due to bottom water temperature increase or depressurization, increases the pore pressure and thus the potential of slope failure (e.g., ref. 35). Our data give first clear evidence for the presence of gas hydrates at the northern Svalbard continental margin.

**Figure 5:**
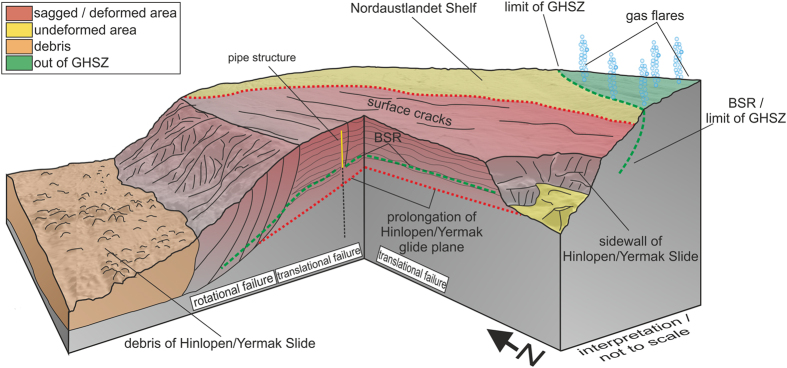
3D conceptual model of the Nordaustlandet Shelf. BSR, Bottom simulating reflector; GHSZ, gas hydrate stability zone.

We interpret the half-bowl-like slope to the north of the HYM sidewalls in accord with the observed curvilinear features along the slope as a result of a sagging of the slope (Fig. 5). Laberg *et al*.^36^ observed similar features offshore northern Norway that are actually cracks in the seafloor and evidence of slope instability. In conjunction with the new seismic data, the steps at the western Nordaustlandet slope (Figs 2 and 3) are interpreted as sediment-covered initially-failed sedimentary blocks in the shallow subsurface associated with evolving, finally not excavated headwalls below. It might be realistic that the sedimentary blocks already slid short distances along shallow glide planes beneath.

The Nordaustlandet lower slope (Figs 2 and 5) shows deformation at listric detachment planes, which are indicators of incipient or arrested rotational block/slide movements (see also refs 23 and 36) (Fig. 5). These glide planes seem to evolve as shear bands out from the internal geometry of the contourite drift deposits, as imaged by our high-resolution seismic data. At the upper slope, the BSR at ~240 m below seafloor presents a potential zone of weakness owing to the reduced shear strength of over-pressured sediments beneath the GHSZ^37^,^38^. Consequently, while the lower slope is dominated by rotational stress, the upper slope could experience translational slope mobilization. Indeed we claim to see the first sights of incipient slope failure, evidenced by internal deformation (folding) of sediments above the major glide plane, sagging of large parts of the slope, newly evolving zones of weakness (glide/shear plans), that could results into new headwalls, and secondary minor slope instabilities at the seafloor above.

The imaged incipiently failed slide block itself is ~240 m thick and its areal extent is ~6 km (the length of the glide plane in the seismic profile, see Fig. 3) by >32 km (length of the bounding N-S step/“crack” in bathymetry, Fig. 1). The area and volume of mobilized sediment are thus ~200 km^2^ and ~50 km^3^, respectively. The mobilized block extends northwards of the HYM sidewalls, implying its mobilization was approximately contemporaneous with the main 30-kyr HYM event or happened shortly afterwards^23^. The total area and volume of the continental slope potentially affected by sediment mobilization could be on the order of 600 km^2^ and 200 km^3^, assuming the height of the HYM’s side walls (~350 m) equals the thickness of its sliding sediments, and taking the bowl-shaped area of the shelf as the maximum lateral extent of mobilization (Fig. 1).

These different components discussed above indicate a potential multi-phase retrogressive slope failure, which began with toe erosion and rotational sliding and was transferred into a translational or block-slide mechanism at the upper slope. As discussed above, pore over-pressure might have played a key role in slope-failure initiation. We might speculate that the release of pore over-pressure through hydro-fracturing and pipe formation actually caused resting of the slope. We do not know if the slope came to rest completely or if it is still deforming slowly by creeping as proposed by Winkelmann *et al*. for the shallow slope sediments^23^. If we accept the available dating for the HYM of ~30000 years, acc. to Winkelmann *et al*.^8^, and assume that the incipient failure at the western Nordaustlandet slope was contemporaneous, then the pore over-pressure could have arisen from methane release from destabilizing gas hydrates at a period of rapid sea-level drop^22^. Under this assumptions gas and gas-overpressure might have indeed played a major role in preconditioning the slope for failure. Similarly, Kvalstad *et al*.^32^ stated that high excess pore pressure also strongly influenced the slide process in the Storegga Slide. Also Vanneste *et al*.^39^ rank excess pore pressure as one of the key parameters for slope instability. We might speculate that the North Spitsbergen slope could be re-activated if gas overpressure reaches again a critically value. We may also speculate that large quantities of methane were released suddenly to the atmosphere during the HYM failure event, if the large now excavated slope areas were also underlain by similar over-pressured gas reservoirs.

The western Nordaustlandet slope still exhibits most of the known prerequisites for a large submarine slide event. The new seismic and bathymetry data imply a potential headwall of ~65–70 km length and up to 350 m height, indicating a large volume (~200 km^3^) would be affected by initial failure. Complete failure would lead to one of the 15 largest non-volcanic submarine slides to have occurred in the past 36000 years^40^. As the affected area is located between the littoral states of the Arctic Ocean, a potential tsunami would not currently affect major coastal populations or infrastructure (see ref. 41). Nevertheless, with on-going sea ice retreat and intensifying arctic exploration, planning for future infrastructure and populations must take the risk of renewed failure at the western Nordaustlandet shelf into consideration. We still do not know what actually triggered the HYM and the initial failure further north and we might only speculate, if and why the not yet failed slope should fail completely. Warming bottom waters could challenge the stability of gas hydrates and therefore the slope stability, but this effect is debated (e.g., ref. 39). Ongoing glacial rebound of eastern Svalbard^42^ could cause local seismic activity, another trigger mechanism. Deep drilling and geotechnical studies would allow proving our interpretations and would strongly help to evaluate the potential future risk of slope failure.

Summarizing, we found evidence of wide-spread methane venting at the Northern Svalbard shelf in close vicinity to the HYM slide scar. Thus, methane emanates from areas where it didn’t or was not noticed before. Furthermore, we found evidence for past (pre-HYM) failure events but also for an incipient major failure of the western Nordaustlandet slope immediately north of the HYM sidewalls. The internal slope deformation follows old sites of weakness such as old slide deposits and old headwalls, and it is also influenced by the internal geometry of contourite deposits. Our new data strongly indicate that gas hydrates, free gas and excess pore pressure may have been triggering or supportive mechanisms for the slope instabilities north of Svalbard.

## Methods

In late summer 2013, a scientific cruise was carried out onboard R/V MARIA S. MERIAN along the northern Svalbard continental margin dedicated to the structure of the shelf in the vicinity of the Hinlopen/Yermak Megaslide. Main objective was to collect new data, to characterize the structural setting and potential trigger mechanisms of the megaslide. This included imaging of ancient and recent fluid systems, gas hydrate indicators, as well as listening for local seismicity.

### Bathymetry

The seafloor was continuously mapped by Kongsberg EM122, and additionally Kongsberg EM1002 in shallow waters. To calibrate the measured depths the sound velocity profile was regularly updated using data from CTD casts. All raw data was processed using the software CARIS HIPS & SIPS. Data was cleaned from coarse errors by using the “Swath Editor”, a ping-by-ping data-cleaning editor and occasionally the “Subset Editor”, where the data can be viewed and cleaned in a 3D-view.

### Water column imaging

Water column and the shallow sub-seafloor were continuously imaged along all track lines using the ATLAS PARASOUND System DS3 (P70) of R/V Maria S. Merian. The system was operated at 18 and 22 kHz, respectively. We used the secondary low frequency (SLF) signal at 4 kHz to image the sediments, and the primary high frequency (PHF) signal at 18 kHz to monitor the water column.

### High-resolution reflection seismic measurements

The 2D reflection seismic system of the Institute of Geosciences, University of Kiel and the GEOMAR Helmholtz-Centre for Ocean Research Kiel was used to acquire high-resolution multichannel seismic data. Using this system we were able to resolve small-scale sedimentary structures and closely spaced layers on a meter scale, which can usually not be resolved by means of conventional seismic systems. During the high-resolution measurements, a 1.7 l GI-Gun and a 0.4 l G-Gun were used as sources. The cruise speed was set to 4.5 knots and a shot interval of 7 s resulting in a shot point interval of ~16 m. Data were recorded with a Geometrics GeoEel digital streamer. The system consists of a tow cable, a 25 m long stretch section, and 10 to 11 active sections of 12.5 m length each. An active section contains 8 channels with a channel spacing of 1.56 m, resulting in 80 to 88 channels within the entire streamer.

The entire dataset was processed by using the commercial software Vista Seismic Processing (Schlumberger). Processing including a 20/40/200/400 Hz band pass filter, despiking, CMP-binning, Normal-Move-Out Correction and debias-filtering. The CMP bin size was set to 3.125 m, which resulted in an average fold of 12. Due to the limited offset range of the relatively short streamer, Normal-Move-Out is not sensitive to a dedicated velocity analysis. Therefore, a constant velocity of 1500 m/s was applied to the dataset, which resulted from the best-fit value during an iterative approach to determine the sound velocities. All data were time-migrated by using the software’s finite difference migration method with a constant velocity of 1500 m/s. All processing steps aimed to result in a reliable illustration of the upper ~1 s TWT beneath the seafloor.

### Water sampling for measurement of gas content

Water sampling and temperature measurements were carried out by a Sea-Bird Electronics Inc. SBE 911plus CTD equipped with a Sea-Bird SBE 32 carousel with 24 Niskin 10l-water samplers.

For DIC measurement at each CTD cast 3 × 100 ml samples were taken from niskin bottles closed at 5 m and 15 m above the seafloor. DIC samples were poisoned with mercury chloride (0.06 mL mercury chloride solution (3.6 g mercury chloride in 100 mL purified water) per 99,994 mL water sample) and stored in waxed 100 mL-borosilicate vials for transport. Immediately after the multicorer was on deck, the tubes were taken out and the water topping the sediment surface was sampled for methane measurements. With aid of two 100 ml syringes equipped with a short tubing the multicorer water was sampled and transferred in 100 mL-borosilicate vials, closed and waxed for transport. For methane measurements water samples were transferred in 20 ml vials, capped with a Teflon septum, and crimped gas tight. A headspace of 2 ml volume was introduced by injecting Argon gas and thereby displacing an equivalent volume of water. After 12 hours of equilibration, the gas concentration in the headspace was analysed with aid of a gaschromatograph TraceGC (Thermo Finnigan; Waltham, USA), equipped with a flame-ionization detector and a Porapak Q column. The GC oven was operated isothermally at 100 °C and the temperature at the sample inlet was 300 °C. Two standard gases (10 ppm and 1000 ppm) were used for the calibration. Based on the methane concentration in the headspace and the methane concentration in the aqueous phase, which was calculated using the Bunsen coefficient^26^,^43^, the methane concentration in the water sample was derived. The overall error of the method was about 5%. Thereafter, methane concentrations from the headspace were measured with a gas chromatograph (GC) (Chrompack, 9003) with flame ionization detector (FID). The standard error of duplicate measurements including both gas extraction and GC analysis was ~5%.

A methane concentration of 152 to 218 nmol/L was measured in the CTD samples 5 m above seafloor. 99 to 103 nmol/L was measured 15.4 m above the seafloor. Water ontop of a multicorer (MUC) sample showed a concentration of 178 nmol/L.

## Additional Information

**How to cite this article**: Geissler, W. H. *et al*. Arctic megaslide at presumed rest. *Sci. Rep.* **6**, 38529; doi: 10.1038/srep38529 (2016).

**Publisher's note:** Springer Nature remains neutral with regard to jurisdictional claims in published maps and institutional affiliations.

## Supplementary Material

Supplementary Figures and Tables
