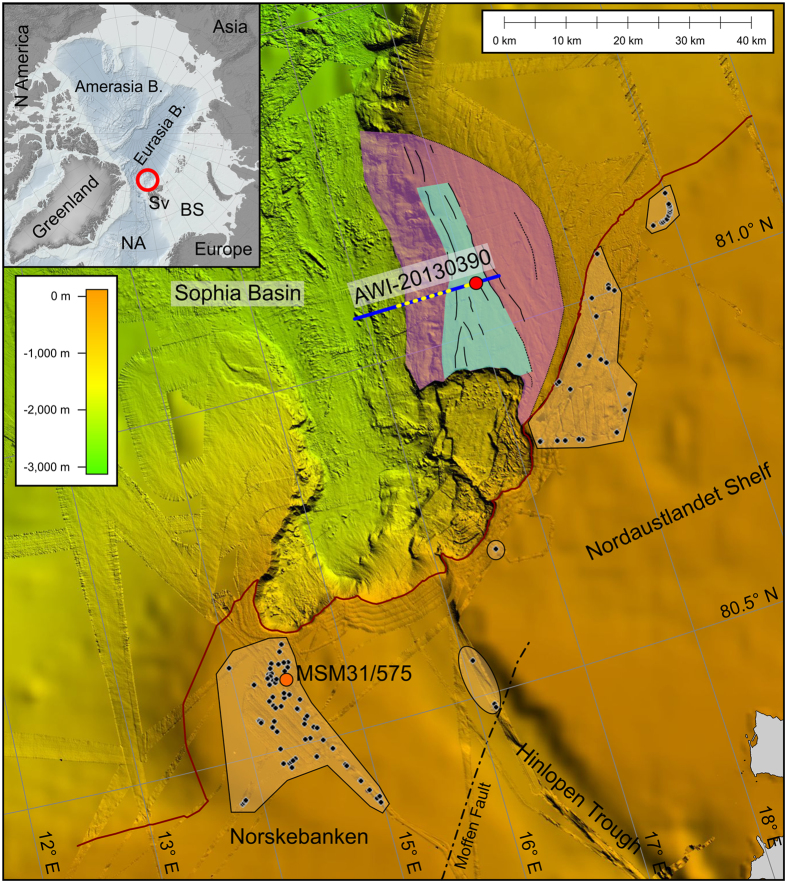# Corrigendum: Arctic megaslide at presumed rest

**DOI:** 10.1038/srep46821

**Published:** 2017-05-17

**Authors:** Wolfram H. Geissler, A. Catalina Gebhardt, Felix Gross, Jutta Wollenburg, Laura Jensen, Mechita C. Schmidt-Aursch, Sebastian Krastel, Judith Elger, Giacomo Osti

Scientific Reports 6: Article number: 3852910.1038/srep38529; published online: 12 06 2016; updated: 05 17 2017

In Figure 1, the latitudes ‘80.5 N’ and ‘81.0 N’ were incorrectly given as ‘81.0 N’ and ‘81.5 N’ respectively. In addition, the scale between 0 km and 40 km was incorrectly given as between 0 km and 80 km. The correct Figure 1 appears below as Figure 1.

## Figures and Tables

**Figure 1:**