# Supplementary material for: Arctic megaslide at presumed rest
Source: Sci Rep. 2016 Dec 6;6:38529. doi: 10.1038/srep38529 (PMC5138642; doi:10.1038/srep38529)
Supplement: Supplementary Figures and Tables [file srep38529-s1.pdf]

Arctic megaslide at presumed rest

Wolfram H. Geissler[1*], A. Catalina Gebhardt[1], Felix Gross[2], Jutta Wollenburg[1], Laura Jensen[1], Mechita C. Schmidt-Aursch[1], Sebastian Krastel[2], Judith Elger[3], Giacomo Osti[4]

[1]Alfred-Wegener-Institut Helmholtz-Zentrum für Polar- und Meeresforschung, Bremerhaven, Germany, contact: Wolfram.Geissler@awi.de.

[2]Christian-Albrechts-Universität, Kiel, Germany.

[3]GEOMAR Helmholtz-Zentrum für Ozeanforschung, Kiel, Germany.

[4]CAGE - Centre for Arctic Gas Hydrate, Environment and Climate, Department of Geology, UiT-The Arctic University of Norway, Tromsø, Norway.

Supplementary Information

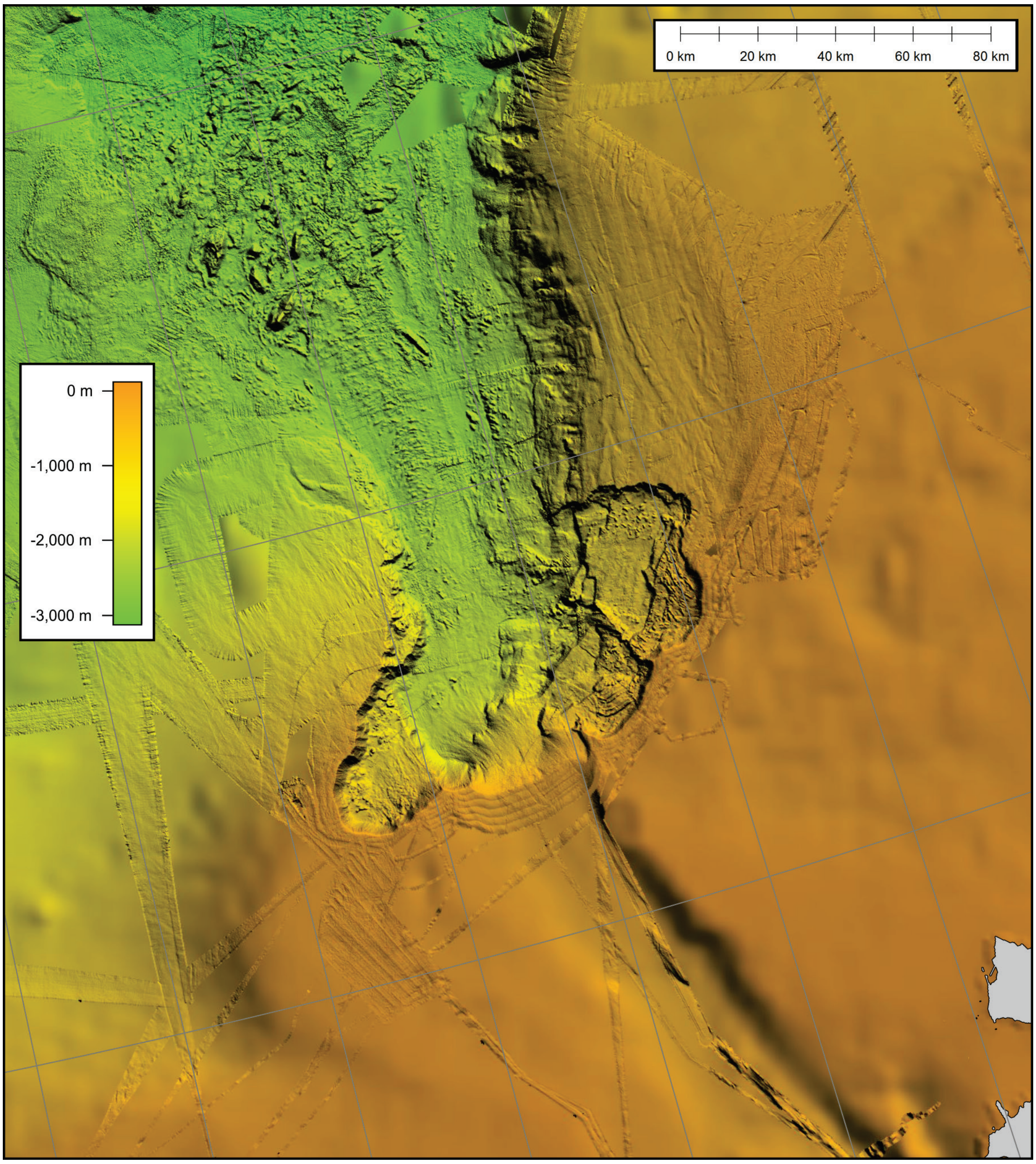

Figure S.1 | Seafloor topography without interpretation. We used Global Mapper (V16.1) to create the map. (sources: Vanneste et al., 2006; Winkelmann et al., 2006; Jakobsson et al., 2012, new data)

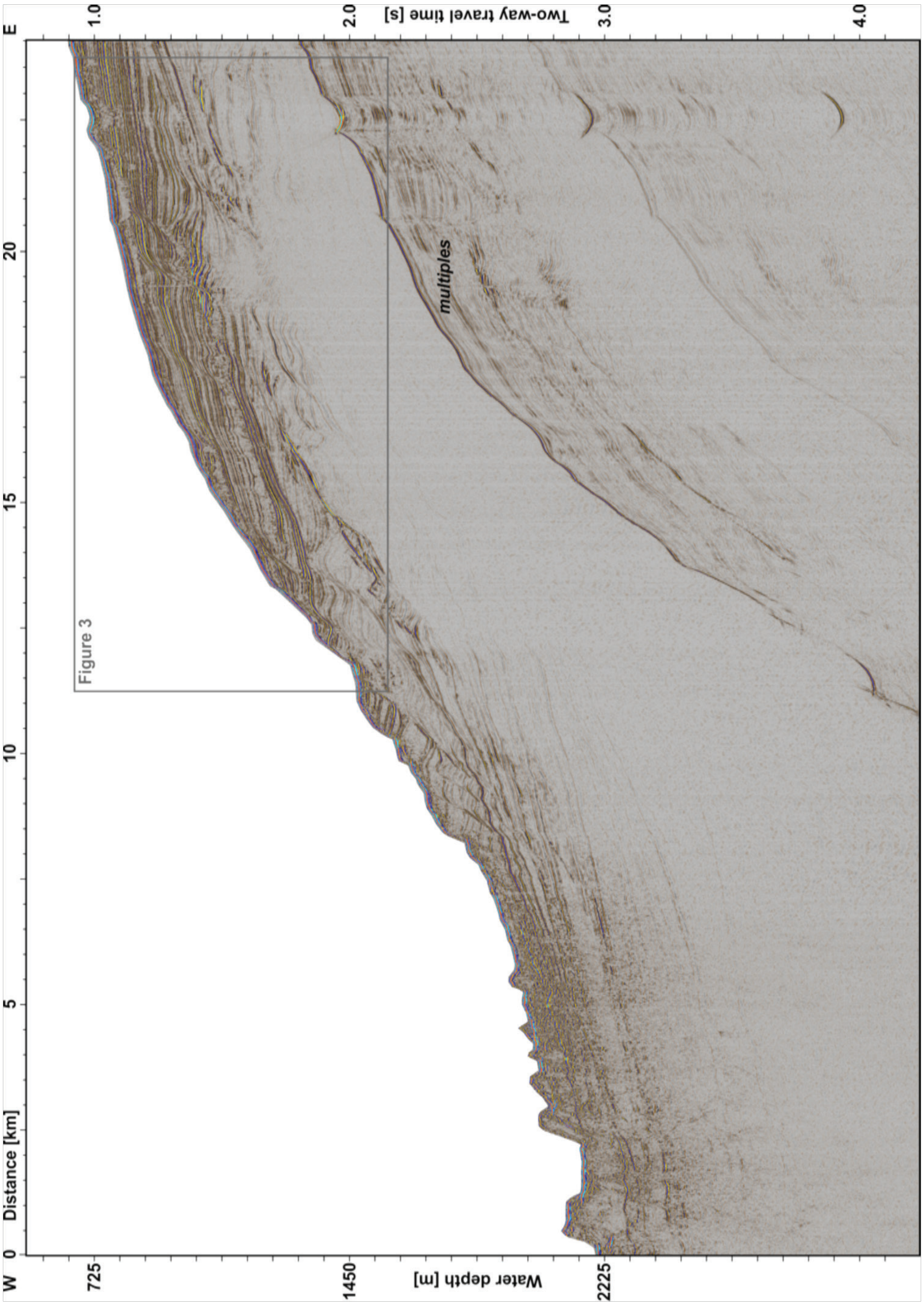

Figure S.2 | Uninterpreted seismic profile AWI-20130390 (see Fig. 2).

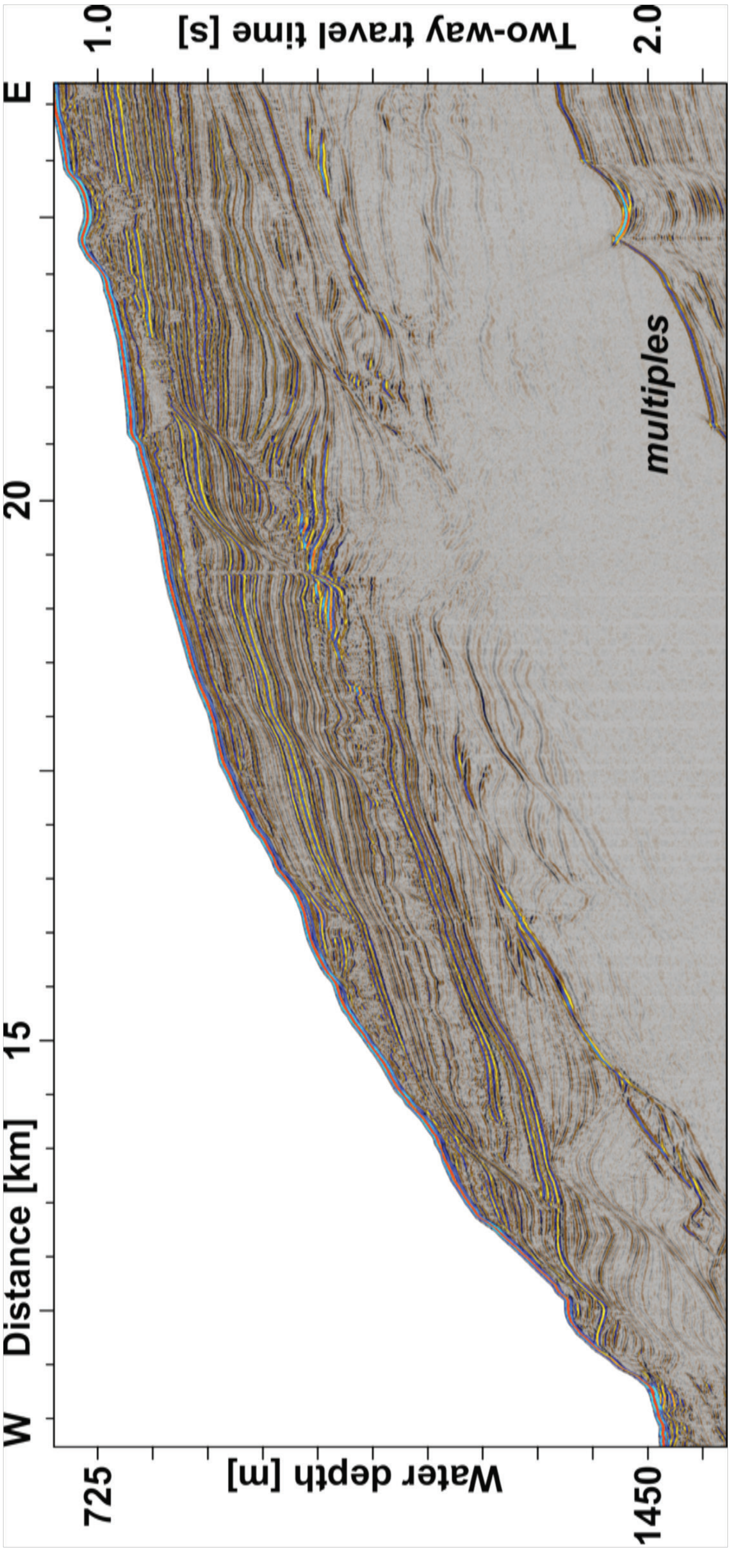

Figure S.3 | Uninterpreted seismic profile AWI-20130390 (detail, see Fig. 3).

Table S.4 | Location of acoustic flares

Date	UTC	Latitude	Longitude
21.08.13	01:30	80.6968	16.5500
22.08.13	12:07	81.0000	18.0500
22.08.13	12:34	81.0000	18.2688
22.08.13	15:57	80.8522	18.1063
22.08.13	16:13	80.8323	18.0188
22.08.13	16:38	80.8052	17.8897
22.08.13	17:16	80.8105	17.5750
22.08.13	17:20	80.8125	17.5448
22.08.13	17:36	80.8167	17.4260
22.08.13	17:45	80.8198	17.3563
22.08.13	18:04	80.8260	17.2147
22.08.13	18:05	80.8260	17.2063
23.08.13	03:43	80.5490	14.1355
23.08.13	03:44	80.5480	14.1417
23.08.13	03:46	80.5468	14.1573
23.08.13	04:27	80.5718	14.3990
23.08.13	04:49	80.5990	14.3510
23.08.13	04:55	80.6063	14.3292
23.08.13	04:56	80.6073	14.3250
23.08.13	04:57	80.6083	14.3218
24.08.13	09:30	81.0708	18.7980
24.08.13	09:39	81.0708	18.8792
24.08.13	09:41	81.0708	18.8958
24.08.13	09:43	81.0718	18.9135
24.08.13	09:45	81.0730	18.9302
24.08.13	09:46	81.0730	18.9375
24.08.13	09:50	81.0772	18.9647
24.08.13	09:53	81.0802	18.9802
24.08.13	09:54	81.0813	18.9843
24.08.13	09:56	81.0843	18.9917
24.08.13	09:58	81.0875	18.9958
24.08.13	09:59	81.0885	18.9968
24.08.13	10:00	81.0897	18.9980
24.08.13	10:02	81.0927	18.9980
24.08.13	10:14	81.1093	18.9990
25.08.13	13:11	80.4385	13.8250
25.08.13	13:13	80.4397	13.8385
25.08.13	13:15	80.4417	13.8522
25.08.13	13:16	80.4427	13.8583
25.08.13	14:19	80.4833	14.3635
25.08.13	14:12	80.4792	14.3032
25.08.13	14:14	80.4802	14.3208
25.08.13	14:16	80.4813	14.3385
25.08.13	15:00	80.5208	14.5855
25.08.13	15:25	80.5510	14.4917
25.08.13	16:02	80.5990	14.3468
25.08.13	16:07	80.6063	14.3260

Date	UTC	Latitude	Longitude
06.09.13	21:34	80.6327	13.9927
06.09.13	22:49	80.6096	14.3168
06.09.13	22:52	80.6072	14.3236
06.09.13	22:58	80.6001	14.3444
06.09.13	23:15	80.5785	14.4078
06.09.13	23:38	80.5521	14.4849
06.09.13	23:52	80.5352	14.5326
07.09.13	00:21	80.4995	14.6374
07.09.13	00:58	80.4572	14.7693
07.09.13	00:59	80.4549	14.7771
07.09.13	01:23	80.4294	14.8607
07.09.13	01:35	80.4130	14.9137
07.09.13	01:48	80.3987	14.9589
07.09.13	01:53	80.3917	14.9823
07.09.13	10:32	80.4007	14.9848
07.09.13	11:25	80.4842	14.7001
07.09.13	12:00	80.5184	14.6126
07.09.13	12:10	80.5384	14.5543
07.09.13	12:27	80.5743	14.4500
07.09.13	12:45	80.6103	14.3454
07.09.13	16:06	80.6472	14.4932
07.09.13	16:49	80.6375	14.4608
07.09.13	16:57	80.6199	14.5104
07.09.13	17:14	80.6130	14.4953
07.09.13	17:17	80.6211	14.4727
07.09.13	17:53	80.6207	14.4237
07.09.13	18:00	80.6089	14.4614
07.09.13	18:08	80.5962	14.4609
07.09.13	18:23	80.6241	14.3644
07.09.13	18:55	80.5952	14.3032
07.09.13	19:30	80.5282	14.5135
07.09.13	20:07	80.5063	14.5328
07.09.13	20:35	80.5612	14.3552
07.09.13	20:43	80.5769	14.3045
07.09.13	21:06	80.6006	14.1708
07.09.13	21:19	80.5722	14.2655
07.09.13	21:23	80.5645	14.2911
07.09.13	21:25	80.5607	14.3040
07.09.13	21:54	80.5067	14.4875
07.09.13	22:09	80.4785	14.5461
07.09.13	23:38	80.5427	14.2638
08.09.13	00:44	80.5274	14.2656
08.09.13	00:48	80.5349	14.2371
08.09.13	01:26	80.5452	14.1497
08.09.13	03:42	80.4723	14.3139
20.08.13	17:54	80.6064	14.3281
20.08.13	17:57	80.5962	14.3577
20.08.13	19:22	80.4288	14.8625
08.09.13	02:24	80.5020	14.2581

Date	UTC	Latitude	Longitude
08.09.13	04:31	80.4805	14.2513
08.09.13	05:58	80.5641	14.5301
08.09.13	06:26	80.6177	14.3443
08.09.13	06:42	80.6048	14.2998
08.09.13	06:48	80.6006	14.3742
09.09.13	18:20	80.8978	17.5118
09.09.13	18:21	80.8995	17.5303
10.09.13	04:42	80.9141	17.7536
10.09.13	09:03	81.0579	18.2233
10.09.13	16:11	81.0100	18.2317
10.09.13	16:12	81.0055	18.2240
10.09.13	16:35	80.9729	18.0380
10.09.13	16:59	80.9219	17.8633
10.09.13	22:42	80.9119	17.9541
11.09.13	00:12	80.9051	17.9935
12.09.13	01:48	80.5523	16.0777
12.09.13	02:17	80.4838	16.1555
12.09.13	02:18	80.4789	16.1607

Table S.5 | Methane concentrations

**Station/cast**	**Water depth**	**CH4 total [nmol/L]**
**MSM31 575-1**	162m/5.4m above ground	180
	162m/5.4m above ground	152
	152/15.4m above ground	99
	152/15.4m above ground	103
**MSM31 575-5**	164m/5m above ground	218
	164m/5m above ground	216
**MSM31 575-2**	MUC core-top water	178

References

Jakobsson, M. et al., 2012, The International Bathymetric Chart of the Arctic Ocean (IBCAO) Version 3.0: Geophys. Res. Lett., v. 39, L12609.

Vanneste, M., Mienert, J., and Bünz, S., 2006, The Hinlopen Slide: A giant, submarine slope failure on the northern Svalbard margin, Arctic Ocean: *Earth Planet. Sci. Lett.,* **v. 245,** p. 373-388.

Winkelmann, D., Jokat, W., Niessen, F., Stein, R., and Winkler, A., 2006, Age and extent of the Yermak Slide north of Spitsbergen, Arctic Ocean: *Geochem. Geophys. Geosys.,* **v. 7,** no. 6, Q06007.
